# Mucinous Cystic Neoplasm of the Pancreas in a Pregnant Woman: A Case Report and Review of the Literature

**DOI:** 10.7759/cureus.37787

**Published:** 2023-04-18

**Authors:** Abdulaziz A Alomair, Rayan A Almohaimeed, Jolan S Alsaud, Daliyah F Alotaibi, Ohud T Alharbi

**Affiliations:** 1 Department of Surgery, King Fahad Specialist Hospital, Qassim, SAU; 2 College of Medicine, Qassim University, Qassim, SAU

**Keywords:** splenectomy, distal pancreatectomy, pregnant woman, pancreas, mucinous cystic neoplasm

## Abstract

Mucinous cystic neoplasms of the pancreas are rare tumors that represent 10% of cystic pancreatic tumors. They are potentially sex hormone-sensitive. However, mucinous cystic neoplasms occurring during pregnancy are relatively uncommon. A 33-year-old woman in her ninth week of gestation was referred to us due to abdominal pain for two months. Magnetic resonance imaging revealed a well-defined unilocular cystic lesion at the tail of the pancreas, measuring 7x6.4 cm. The patient underwent tumor resection with distal pancreatectomy and splenectomy during the second trimester to prevent the potential risk of rupture of the neoplasm, rapid growth, and/or intrauterine growth restriction. Histopathological examination revealed a mucinous cystadenoma with no atypia or malignancy. The patient completely recovered from the surgery and had a healthy full-term baby. This case shows the benefit of performing the surgery during the second trimester compared to the potential risk of delaying the surgery.

## Introduction

About 10% of cystic pancreatic tumors are mucinous cystic neoplasms (MCN), a rare type of cystic neoplasm of the pancreas. Approximately 93%-95% of these tumors are located in the body and tail of the pancreas. [1-3] The presence of mucin-producing columnar epithelium supported by an ovarian-type stroma serves as their primary identifying characteristic. Estrogen and progesterone receptors are expressed in the ovarian-type stroma of pancreatic MCN, suggesting that female sex hormones may impact pancreatic MCN activity, particularly during pregnancy. [4-7] However, a few reports have published MCN occurring during pregnancy with surgical resection before delivery (Table 1). This report presents the case of a 33-year-old pregnant woman with a mucinous cystic neoplasm at the tail of the pancreas. Also, it shows the benefit of performing the surgery during the second trimester compared to the potential risk of delaying the surgery.

## Case presentation

A 33-year-old woman, gravida 6, para 4+1, presented in her ninth week of pregnancy with recurrent abdominal pain for two months. The abdominal pain was aggravated by spicy foods and was associated with nausea, vomiting, and shortness of breath, with no fever, urinary symptoms, or melena. She is not known to have any chronic illnesses.

**Figure 1:**
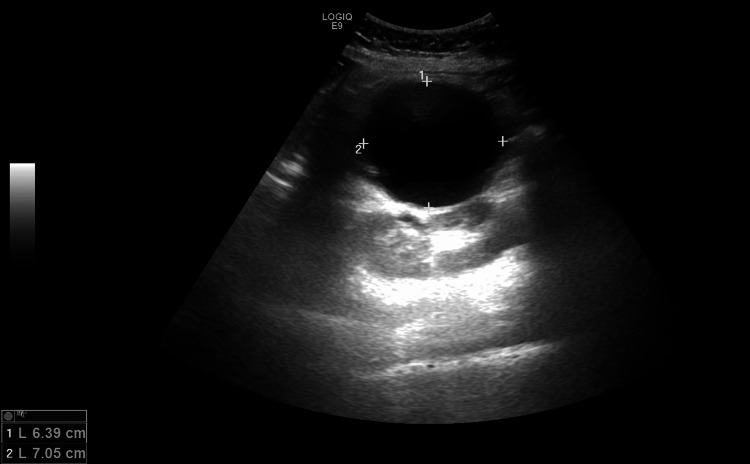
An abdominal ultrasound demonstrating a 7 x 6.4 cm well-defined anechoic cyst in the region of the pancreatic tail.

Clinical examination shows epigastric mass and tenderness. Abdominal ultrasound revealed a well-defined anechoic cyst in the region of the pancreatic tail measuring 7x6.4 cm (Figure 1).

**Figure 2:**
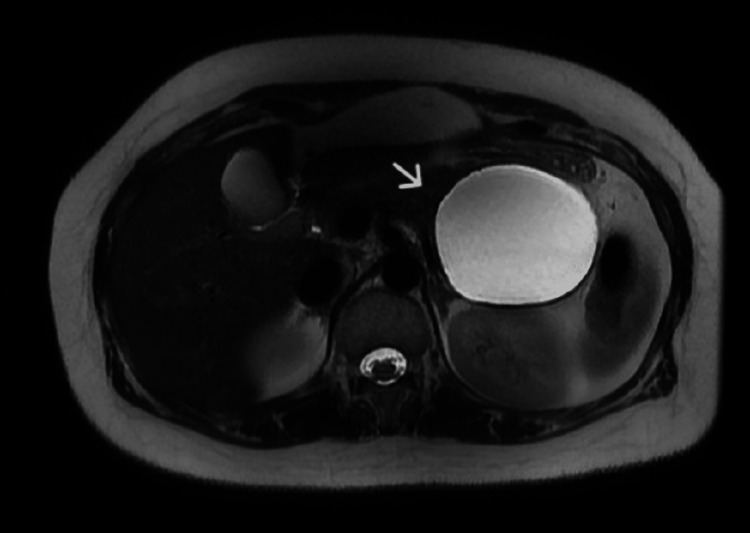
Magnetic resonance imaging revealed a well-defined unilocular cystic lesion at the tail of the pancreas, which, on histopathological examination, demonstrated an MCN.

Further evaluation with magnetic resonance imaging (MRI) revealed a well-defined unilocular cystic lesion at the tail of the pancreas without an established pancreatic duct connection, which may represent a mucinous neoplasm (Figure 2).

Her laboratory investigations showed normal liver enzymes and amylase levels with mild anemia. The serum levels of tumor markers carcinoembryonic antigen (CEA), cancer antigen 15-3 (CA15-3), and cancer antigen 125 (CA-125) were within normal ranges, and cancer antigen 19-9 (CA 19-9) was normal. The patient was informed regarding the potential risks of malignancy, pancreatitis, rupture of the neoplasm, rapid growth, compressing surrounding tissues, and/or intrauterine growth restriction. Therefore, resection was planned during the second trimester, a period in which the risks were considered to be the lowest for both the patient and the fetus.

**Figure 3:**
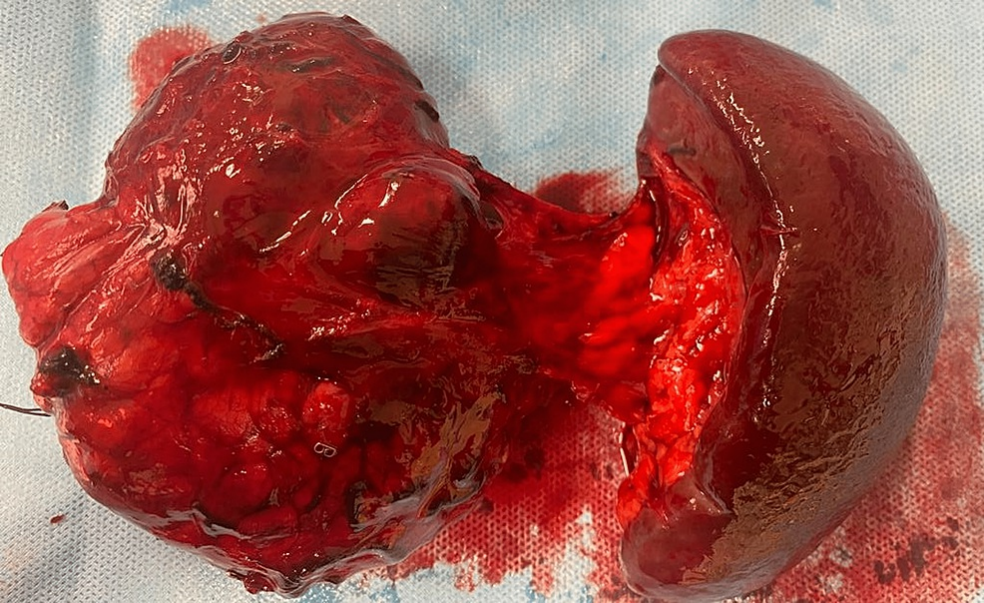
Intraoperative finding and macroscopic view of the resected tumor and spleen

The patient underwent laparotomy for tumor resection at 20 weeks gestation. Distal pancreatectomy and splenectomy were performed (Figure 3).

**Figure 4:**
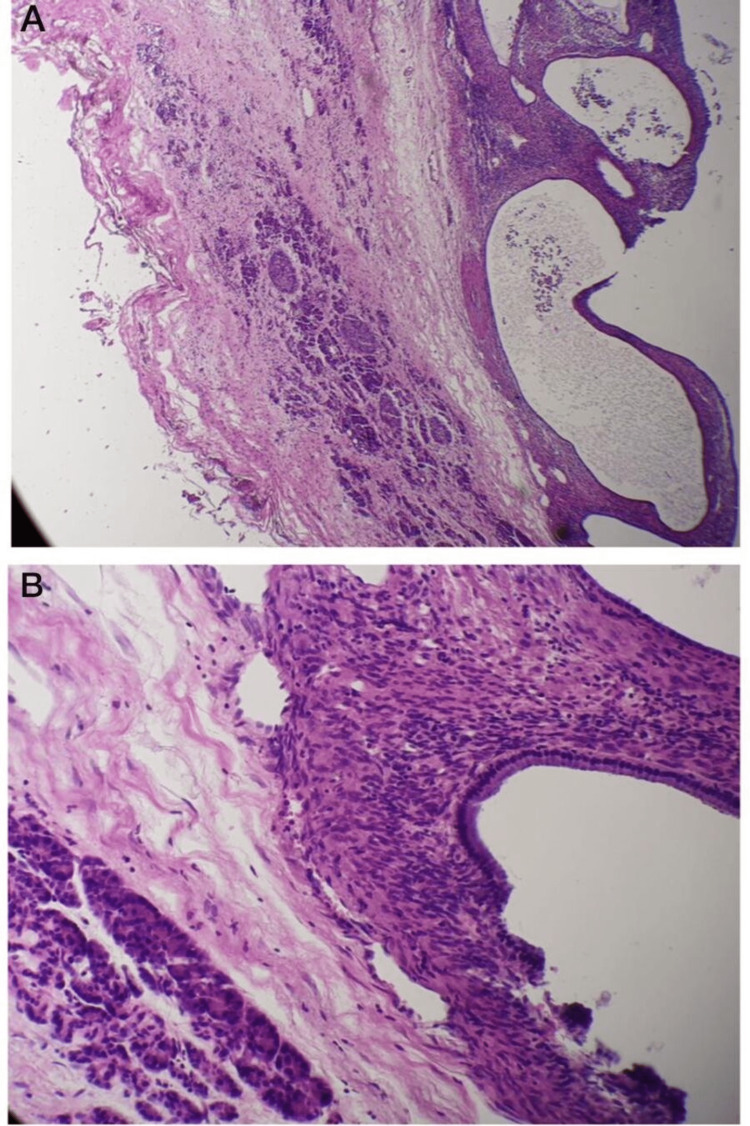
Histopathological examination of the resected pancreatic cystic neoplasm. (A) Pancreatic tissue with a cystic lesion. (B) Part of the cystic lesion shows mucinous lining epithelium with no atypia or malignancy. These findings led to the diagnosis of mucinous cystadenoma.

Histopathological examination revealed a mucinous cystadenoma with a 0.5 cm safety resection margin and one reactive lymph node (Figure 4). No atypia or malignancy was seen in the examined section.

The abdominal symptoms improved, and the postoperative course was uneventful. Postoperative follow-ups after one and six months showed no abnormalities during the clinical examination, blood tests, or abdominal ultrasonography (USG). The patient had a healthy full-term baby through a cesarean section and will maintain regular follow-ups at the general surgery department.

## Discussion

It is uncommon for a pancreatic neoplasm to develop in a young patient, and it is rare for MCN to be associated with pregnancy (Table 1).

**Table 1:** Summary of previously reported pancreatic MCNs associated with pregnancy with surgical resection before delivery DP: distal pancreatectomy; NA: not available; NR: not reported; IUGR: intrauterine growth restriction

No.	Author/Citation	Age at diagnosis	Gestational age at diagnosis (weeks)	Timing of operation	Complications	Surgical procedure	Histological diagnosis
1	Olsen [11]	25	5	Week 18	NR	DP	Adenoma
2	Ganepola [12]	37	4	Week 23	NR	DP	Adenoma
3	Kato [6]	33	15	Week 23	IUGR	DP	Adenoma
4	Fernández [5]	26	20	Week 26	Episodic epigastric pain	DP	Adenoma
5	Herring [13]	34	3	Week 17	NR	DP	Adenocarcinoma
6	Hakamada [14]	38	Two years before pregnancy	Second trimester	nausea, hematemesis, tarry stool	DP, partial stomach resection	Borderline
7	Wiseman [15]	32	11	Week 15	Intractable nausea	DP	Low-grade dysplasia
8	Brown [16]	38	8	Week 8	Gastrointestinal bleeding	DP	Severe dysplasia
9	Martins-Filho [17]	20	20	Week 20	No	DP	Adenoma
10	Boyd [18]	21	Seven months before pregnancy	Week 20	Abdominal distension and fullness	DP	Moderate dysplasia
11	Tsuda [19]	28	Some years before	Week 18	No	DP	Severe dysplasia
12	Veits [20]	28	11	NA	Pancreatitis	DP	Low-grade dysplasia
13	Revoredo [21]	38	17	Week 29	Rupture	DP	Moderate dysplasia
14	Revoredo [21]	30	18	Week 20	No	DP	Adenocarcinoma
15	Present case	33	9	Week 20	Episodic epigastric pain	DP	Adenoma

MCNs are benign in most cases and usually grow slowly. However, the majority of case reports mention notably rapid growth and larger size during pregnancy, which may also raise the risk for malignant transformation into invasive carcinomas, pancreatitis, compressing surrounding tissues, and potentially leading to tumor rupture or fetal hazards such as intrauterine growth restriction. [5-8]

The previous study shows that pregnant women have been represented by a larger pancreatic MCN than the non-pregnant population. [9] The reason may be due to expressed estrogen and progesterone receptors in the ovarian-type stroma of pancreatic MCN, suggesting that female sex hormones may impact pancreatic MCN activity, particularly during pregnancy. [4-7]

Abdominal USG, MRI, CT, and eventually endoscopic ultrasound sonography (EUS) with fine needle aspiration (FNA) are required to assess and evaluate pancreatic MCN before making any diagnosis or management. However, CT scans should be avoided during pregnancy due to the potential risk of fetal malformation. In addition, according to the European evidence-based guidelines on managing cystic pancreatic neoplasms (2018), if a clear surgical justification exists, EUS with FNA is not recommended. [10] Therefore, MRI is the best investigation for pancreatic MCNs during pregnancy.

Unfortunately, there are no standards for managing pancreatic MCNs during pregnancy, and the decision is based on expert opinions. There are many factors to consider, including malignant predictors, fetal-maternal impairment, and gestational age. For our patient, we decided to perform surgical excision of the tumor mass in the second trimester, considered the safest surgery period for both the patient and fetus, to prevent any adverse effects that may happen if we delay the surgery until after delivery.

## Conclusions

Mucinous cystic neoplasms occurring during pregnancy are relatively uncommon. MCNs are benign in most cases and usually grow slowly. However, most case reports mention rapid growth and larger size during pregnancy, which may also raise the risk for malignant transformation into invasive carcinomas, pancreatitis, compressing surrounding tissues, and potentially leading to tumor rupture or fetal hazards such as intrauterine growth restriction. Therefore, we recommend a surgical resection during the second trimester, a period in which the risks are considered to be the lowest for both the patient and fetus, to prevent any adverse effects that may happen if surgery is delayed until after delivery.

## References

[1] Nilsson LN, Keane MG, Shamali A (2016). Nature and management of pancreatic mucinous cystic neoplasm (MCN): a systematic review of the literature. Pancreatology.

[2] Dhali A, Ray S, Khamrui S, Dhali GK (2021). Mucinous cystadenocarcinoma of pancreas mimicking gastrointestinal stromal tumor of stomach: case report. Int J Surg Case Rep.

[3] Miller FH, Lopes Vendrami C, Recht HS (2022). Pancreatic cystic lesions and malignancy: assessment, guidelines, and the field defect. Radiographics.

[4] Xie W, Liang H, Guo Y, Xiao SY (2021). Update on mucinous cystic neoplasm of the pancreas: a narrative review. Journal of Pancreatology.

[5] López-Tomassetti Fernández EM, Martín Malagón A, Arteaga Gonzalez I, Muñiz Montes JR, Díaz Luis H, González Hermoso F, Carrillo Pallares A (2005). Mucinous cystic neoplasm of the pancreas during pregnancy: the importance of proper management. J Hepatobiliary Pancreat Surg.

[6] Kato M, Kubota K, Kita J (2005). Huge mucinous cystadenoma of the pancreas developing during pregnancy: a case report. Pancreas.

[7] Naganuma S, Honda K, Noriki S (2011). Ruptured mucinous cystic neoplasm with an associated invasive carcinoma of pancreatic head in a pregnant woman: report of a case and review of literature. Pathol Int.

[8] Esposito I, Schlitter AM, Sipos B, Klöppel G (2015). Classification and malignant potential of pancreatic cystic tumors [Article in German]. Pathologe.

[9] Yamao K, Yanagisawa A, Takahashi K (2011). Clinicopathological features and prognosis of mucinous cystic neoplasm with ovarian-type stroma: a multi-institutional study of the Japan pancreas society. Pancreas.

[10] European Study Group on Cystic Tumours of the Pancreas (2018). European evidence-based guidelines on pancreatic cystic neoplasms. Gut.

[11] Olsen M, Greer M, Feintuch T (1993). Pancreatic mucinous cystadenoma during pregnancy. Am J Gynecol Heal.

[12] Ganepola GA, Gritsman AY, Asimakopulos N, Yiengpruksawan A (1999). Are pancreatic tumors hormone dependent?: a case report of unusual, rapidly growing pancreatic tumor during pregnancy, its possible relationship to female sex hormones, and review of the literature. Am Surg.

[13] Herring AA, Graubard MB, Gan SI, Schwaitzberg SD (2007). Mucinous cystadenocarcinoma of the pancreas during pregnancy. Pancreas.

[14] Hakamada K, Miura T, Kimura A, Nara M, Toyoki Y, Narumi S, Sasak M (2008). Anaplastic carcinoma associated with a mucinous cystic neoplasm of the pancreas during pregnancy: report of a case and a review of the literature. World J Gastroenterol.

[15] Wiseman JE, Yamamoto M, Nguyen TD, Bonadio J, Imagawa DK (2008). Cystic pancreatic neoplasm in pregnancy: a case report and review of the literature. Arch Surg.

[16] Brown TH, Menon VS, Richards DG, Griffiths AP (2009). Gastrointestinal bleeding in a pregnant woman: mucinous cystic neoplasm of pancreas mimicking gastrointestinal stromal tumor of stomach. J Hepatobiliary Pancreat Surg.

[17] Filho EDm, Lima T, Jaques RPM, Guerra GV, Adeodato L, Kreimer F (2011). Neoplasia cística mucinosa do pâncreas durante a gestação: relato de caso [Article in Spanish]. Rev Bras Saude Materno Infant.

[18] Boyd CA, Benarroch-Gampel J, Kilic G, Kruse EJ, Weber SM, Riall TS (2012). Pancreatic neoplasms in pregnancy: diagnosis, complications, and management. J Gastrointest Surg.

[19] Tsuda H, Kotani T, Sumigama S, Mano Y, Shimoyama Y, Kikkawa F (2012). Mucinous cystic neoplasm of the pancreas with severe dysplasia during pregnancy: case report and review of the literature. Taiwan J Obstet Gynecol.

[20] Veits L, Deuerling J, Henneking K, Falkeis C, Sterlacci W, Vieth M (2015). Ungewöhnlicher Fall einer muzinösen zystischen Neoplasie des Pankreasschwanzes bei einer schwangeren Frau [Article in German]. Endosk Heute [Internet.

[21] Revoredo F, de Vinatea J, Reaño G, Villanueva L, Kometter F, Arenas J, Polanco PM (2020). Mucinous cystic neoplasms of the pancreas associated with pregnancy: two case reports. Medicine (Baltimore).

